# Supporting bereavement and complicated grief in primary care: a realist review

**DOI:** 10.3399/BJGPO.2021.0008

**Published:** 2021-04-14

**Authors:** Caroline Pearce, Geoff Wong, Isla Kuhn, Stephen Barclay

**Affiliations:** 1 Research Associate, Primary Care Unit, Department of Public Health and Primary Care, University of Cambridge, Cambridge, UK; 2 General Practitioner and Associate Professor, Nuffield Department of Primary Care Health Sciences, University of Oxford, Oxford, UK; 3 Head of Medical Library Services, University of Cambridge Medical Library, University of Cambridge, Cambridge, UK; 4 GP and University Senior Lecturer, Primary Care Unit, Department of Public Health and Primary Care, University of Cambridge, Cambridge, UK

**Keywords:** bereavement, grief, complicated grief, general practice, primary health care, community nursing

## Abstract

**Background:**

Bereavement can have significant impacts on physical and mental health, and a minority of people experience complicated and prolonged grief responses. Primary care is ideally situated to offer bereavement care, yet UK provision remains variable and practitioners feel uncertain how best to support bereaved patients.

**Aim:**

To identify what works, how, and for whom, in the management of complicated grief (CG) in primary care.

**Design & setting:**

A review of evidence on the management of CG and bereavement in UK primary care settings.

**Method:**

A realist approach was taken that aims to provide causal explanations through the generation and articulation of contexts, mechanisms, and outcomes.

**Results:**

Forty-two articles were included. Evidence on the primary care management of complicated or prolonged grief was limited. GPs and nurses view bereavement support as part of their role, yet experience uncertainty over the appropriate extent of their involvement. Patients and clinicians often have differing views on the role of primary care in bereavement. Training in bereavement, local systems for reporting deaths, practitioner time, and resources can assist or hinder bereavement care provision. Practitioners find bereavement care can be emotionally challenging. Understanding patients’ needs can encourage a proactive response and help identify appropriate support.

**Conclusion:**

Bereavement care in primary care remains variable and practitioners feel unprepared to provide appropriate bereavement care. Patients at higher risk of complicated or prolonged grief may fail to receive the support they need from primary care. Further research is required to address the potential unmet needs of bereaved patients.

## How this fits in

Bereavement can lead to prolonged and complicated grief responses impacting on physical and mental health. Evidence from this review shows that bereavement care is considered an important part of primary care, although the way in which it is provided remains inconsistent, and clinicians experience many ambiguities as to the appropriate extent of their involvement with bereaved patients. Following bereavement, patients expect acknowledgement from their GP; however, clinicians often feel unprepared and lack the appropriate resources or training to provide bereavement support. Broader concerns over clinical intervention into bereavement mean bereavement care is not always recognised as a legitimate part of general practice. Awareness of CG among GPs appears low, indicating a gap in education and training in this area.

## Introduction

Most people adapt to bereavement without formal support. However, a minority of bereaved people develop complicated or prolonged grief symptoms, experiencing disruption in daily functioning.^1,2^ Complicated grief (CG) or Prolonged grief disorder (PGD) is a mental health condition involving a pervasive grief response that persists for more than 6 months following a loss.^3^ Patients experiencing severe grief responses may benefit from healthcare support and onward referral for targeted treatment.^4–7^

GPs are frequently identified as ideally placed to offer bereavement support,^8,9^ yet primary care practitioners are often uncertain how to best support bereaved people^10^ and awareness of CG or PGD is low.^11^ In the UK, there remains no consistent approach to general bereavement care,^4^ or to managing severe grief symptoms, despite protocols for general practice proposed over 20 years ago.^8^ NHS policy recognises the importance of bereavement care,^12^ but nationally provision is varied.^13–16^

The COVID-19 pandemic has highlighted the need to improve support for bereaved patients;^17^ it is predicted that circumstances related to the pandemic will increase the numbers of people at risk of complicated grieving.^18–21^ Evidence to inform practitioners and policymakers is vital to ensure bereaved patients are supported over the immediate and longer-term impact.

The authors sought to review the existing evidence to identify what works, how, and for whom in the management of CG in primary care, focusing on the implications for the UK.

## Method

A realist approach was used to conduct this review, as detailed in the published protocol.^22^ Realist review is an interpretive, theory-driven approach to evidence synthesis that is rooted in the principles of ‘realism’, a philosophy of science. Realist reviews build explanations for outcomes that take the form of context–mechanism–outcome configurations (CMOCs).^23^ This approach maintains that aspects of interventions can function as a context to influence the responses of participants (mechanisms) that cause particular outcomes, and is suitable for understanding complex social problems, such as bereavement, where interventions involve multifaceted processes. Box 1 details the review stages based on Pawson’s^23^ protocol. For further details, see Supplementary Box 1.

Box 1 **Realist review stages**

**Table:** 

Step 1. Locating existing theories	An initial programme theory was devised, drawing on the knowledge of the research team. To identify the focus of the review, an exploratory search was conducted using keywords in MEDLINE/PubMed and Google Scholar. Following initial searches, the limited evidence on the management of complicated grief in primary care settings in the UK and other settings was apparent, and thescope of the searches broadened to include general bereavement care.
Step 2. Searching for evidence	The search strategy was conducted with the support of an information specialist (IK). The main search strategy used combinations of search terms for the concepts 'bereavement’, ‘complicated grief’, ‘primary care’, and ‘United Kingdom’ to run four searches (see Supplementary Box S2). In August 2019 the following databases were searched: MEDLINE, Embase, CINAHL (the Cumulative Index to Nursing and Allied Health Literature), PsycINFO, Web of Science, Scopus, ASSIA (Applied Social Sciences Index and Abstracts), Sociological abstracts and SCIE (Social Care Institute for Excellence) Social Care Online.Citation chasing and manual searches of the reference lists of articles and reports were also completed.Following initial screening, the limited evidence on the management of complicated grief in UK primary care was apparent. Therefore, an additional search using a refined search filter for the United Kingdom was undertaken in January 2020 to ensure all relevant UK literature was retrieved.
Screening	Titles and abstracts were screened by CP against the inclusion and exclusion criteria. Inclusion criteria were studies of: (1) adults (aged >18 years) who have lost a significant other and whose grief and/or bereavement experience is considered complicated or prolonged in accordance with a variety of criteria (formal diagnosis of ICD/DSM [International Classification of Diseases/the Diagnostic and Statistical Manual of Mental Disorders] or other not required); and (2) where care is sought and received in primary care settings, focusing on the UK but also including studies from other high and middle income countries where psychosocial conditions can, routinely, be managed in primary care. All study designs were included. Exclusion criteria were studies of neo- and peri-natal bereavement, bereaved children and adolescents, military bereavement, non-death bereavement, grief of healthcare professionals.Judgments on the relevance of the data within these articles for programme theory development were reviewed by the whole project team.
Step 3. Selecting articles	Full-text documents were selected for inclusion based on their ability to provide relevant data to the review. This included articles that directly investigated, reviewed, and discussed how bereavement and complicated grief is managed in primary care settings, in the UK and elsewhere. CP read the full texts and classified them into categories of high and low relevance, based on her judgments on the relevance of the data within these articles for programme theory development. These decisions were then reviewed by SB and GW.At the point of data inclusion based on relevance, when needed, the trustworthiness and rigour of the methods used in each study to gather relevant data was also assessed. One means of assessing rigour was examining whether the study methods had been clearly explained and justified. For example, if using questionnaires or diagnostic tools, then the trustworthiness of data would be considered greater if the questionnaire or tool had been tested and validated.A range of articles were included in the synthesis, including empirical studies, grey literature, and non-empirical articles such as editorials and opinion pieces. Articles were not excluded on the basis of rigour alone if the article contained data relevant to the development of the programme theory.Realist reviews are a type of configurational review, where data are interpreted and used to develop theoretical explanations of phenomena, in this case bereavement and complicated grief in primary care. To build these explanations, even less rigorous data can be useful. Assessments of rigour of included data tend to be undertaken when there is a paucity of data to inform context–mechanism–outcome configurations (CMOCs). It is also equally, if not more, important to judge the explanatory powers of any theories developed, and this was done in the review using the criteria of consilience, simplicity, and analogy at the level of the CMOC and programme theory. A more in-depth explanation and justification of the approach may be found in Wong 2018.^85^
Step 4. Extracting and organising data	The articles were coded in NVivo (version 12), by CP, and initial codes were discussed and refined through iterative discussions with GW and SB. As analysis continued, a refined version of the programme theory was developed. Codes were developed by referring back to the refined programme theory and codes were applied deductively with new codes created as needed (inductively).A realist logic of analysis was applied to the data and sections of texts found within each broad conceptual theme.
Step 5. Synthesising the evidence and drawing conclusions	Working across and within coded-data extracts, CMOCs were developed as part of an iterative development of causal explanations. This included comparing and contrasting data presented in different articles, and making judgments about whether similarities between findings presented in different sources were adequate to form patterns in the development of CMOCs and programme theory.The final product of this stage was a refined realist programme theory that explained how bereaved patients are identified and managed in primary care. The programme theory formed the basis of the implications for practice.

Patient and public members, and stakeholders provided feedback and advice on the review findings. These were incorporated into the development of the final programme theory and helped shape the final recommendations on primary care interventions.

## Results

**Figure 1.**
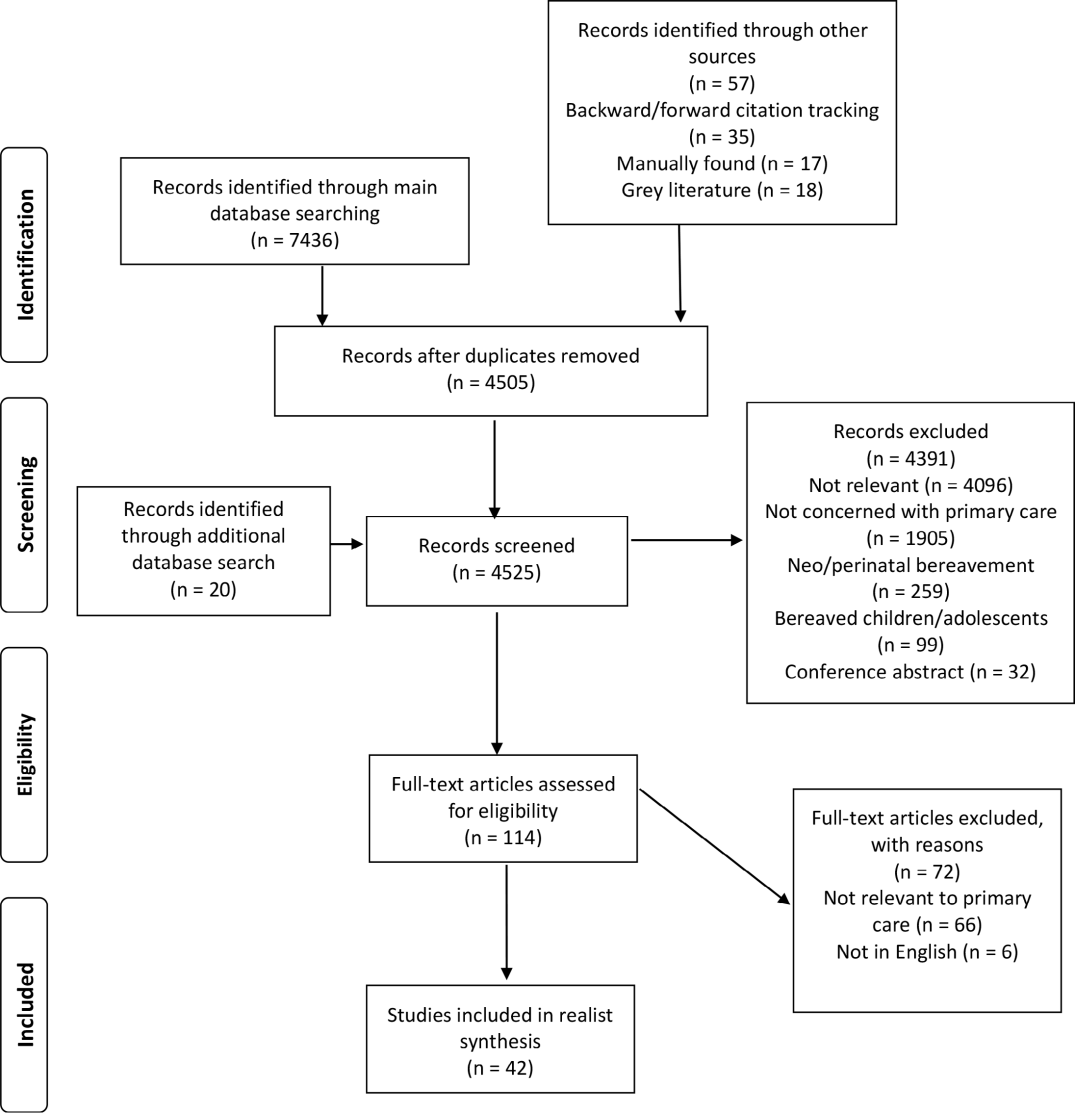
Article selection process

Forty-two articles were included in the analysis (see Figure 1). Most articles reported research (*n* = 35); three commentaries and four letters were also included. Research articles comprised 15 qualitative studies, 11 surveys, six mixed-methods studies, two randomised controlled trials, and one systematic review. Full details are provided in Supplementary Table 1.

No UK articles were found on primary care management of complicated or prolonged grief. Outside the UK evidence was also limited, with only three articles identified that included analysis of practitioner understanding of PGD or CG.^24–26^

**Figure 2.**
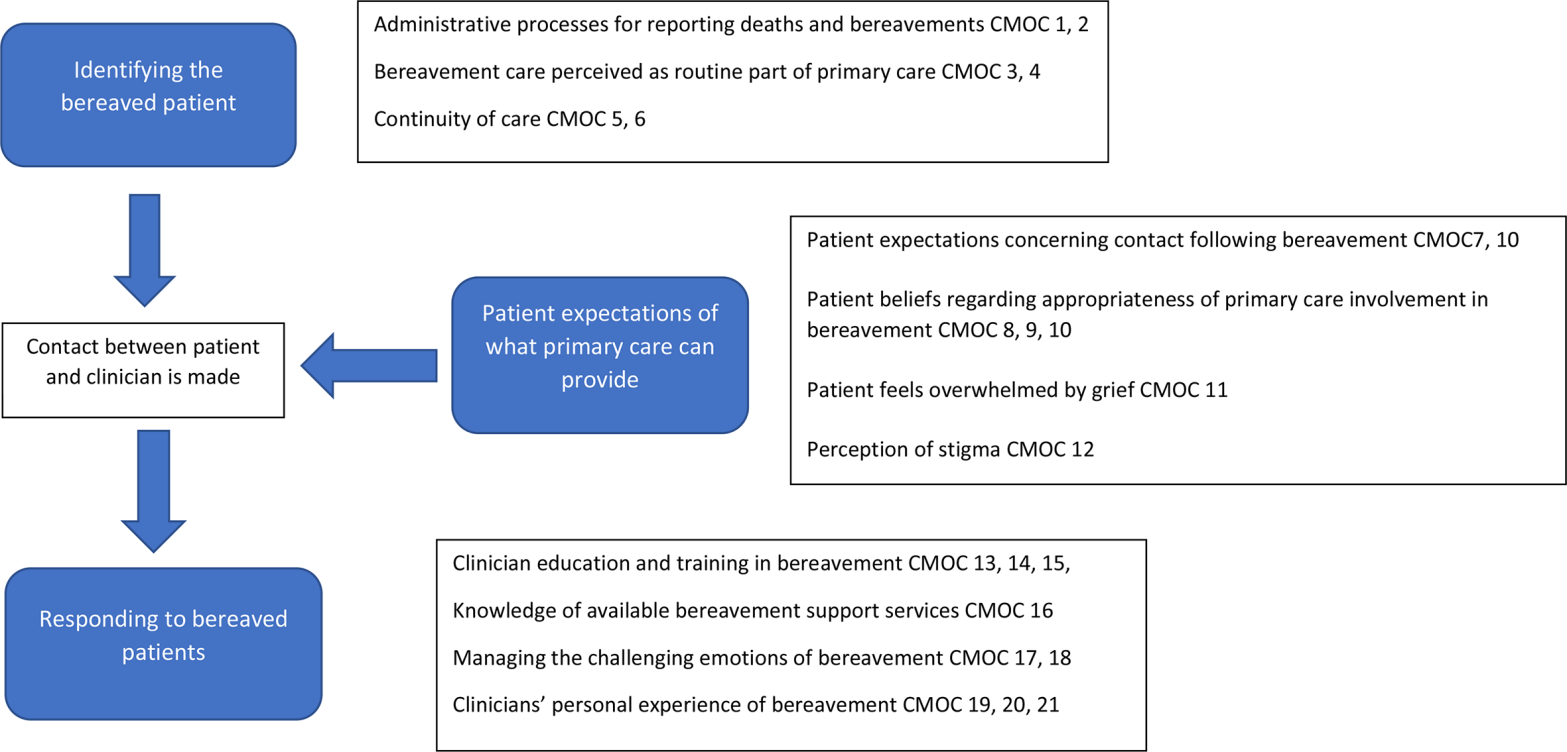
Programme theory of supporting bereavement and complicated grief in primary care. CMOC = context–mechanism–outcome configurations

Broadening the review to general bereavement care, it was found that primary care faced challenges in three areas: identifying bereaved patients; bereaved patients’ expectations; and responding to bereaved patients. In the following sections, a narrative explanation for each issue is provided, referenced with the related CMOCs. Example CMOCs for each area are listed in Table 1; the full list of 21 CMOCs with examples of supporting data are provided in Supplementary Table 2. The final programme theory is presented in Figure 2.

**Table 1.** Themes with accompanying examples of context–mechanism–outcome configurations

**Theme**	**Example context–mechanism–outcome configurations (CMOCs**)
Identifying bereaved patients	If there are no systematic and consistent processes for recording deaths and bereavement (C) clinicians may not be able to identify the bereaved patient (O), because of a lack of awareness (M) (CMOC1)When clinicians have a pre-existing knowledge about the preferences of a recently bereaved person (C), they are more likely to make contact (O), because of their familiarity [of their preference] (M) (CMOC5)
Bereaved person’s expectations of what primary care can provide	When a patient has a bereavement (C), most will expect to be contacted after a bereavement (O), because they believe clinicians are meant to care for them in the hour of their need (M) (CMOC7)When a person is suffering from bereavement (C), they may be unable to seek help or ask for help (O), because they feel overwhelmed (M) and/or hopeless (M) (CMOC11)
Responding to bereaved patients	When clinicians have had limited education in the diagnosis and management of bereavement (C), they find dealing with such patients challenging (O), because of their knowledge gaps (M) (CMOC13)Whether or not professional training has been undertaken, when dealing with patient bereavement (C) clinicians find the experience uncomfortable or unpleasant (O) because such encounters can be emotionally charged and distressing (M) (CMOC17)When clinicians have personal and experiential knowledge of bereavement (C) they have a better understanding of how to manage bereaved patients (O), because they can empathise (M) and appreciate (M) their needs (CMOC19)
For full list of CMOCs with accompanying quotes see Supplementary Table S2	

### Identifying bereaved patients

Inconsistent systems and processes to report deaths were described (CMOC1).^9,27,28^ Death registers improved awareness but difficulties were encountered if the deceased person was registered at a different practice to the bereaved patient (CMOC2).^29,30^ Learning of deaths could be opportunistic.^31^ GPs reported feeling let down by services or processes if unaware their patients had experienced a recent bereavement (CMOC2).^9,24,31^

Most GPs and district nurses considered bereavement support part of caring for the overall health of their patients, and their professional responsibility (CMOC3).^31–34^ When the deceased person was their patient, bereavement support was viewed as an extension of that care.^35,36^ A sense of uncertainty over the appropriate extent of clinician involvement in bereavement was also reported (CMOC3).^31,37–40^ Bereavement care involvement depended not only on perception of role, but also clinicians’ capacity.^33,41^ GPs described lack of time as a primary barrier to engagement with bereavement (CMOC4).^24,27,41^

Continuity of care and a deeper involvement with patients increased likelihood of a proactive response to bereavement (CMOC5).^9,27,35,36,39,40,42^ Greater knowledge of patient preferences and needs could allow the clinician to target support and provide support on an individual level (CMOC6).^31,43^ For example, clinicians considered a patient’s family and social support when judging how a patient was coping.^44^ Patients also felt bereavement support was most helpful when the staff member was known to them.^45^ For those who did not have a strong relationship and/or little knowledge of the bereaved patient’s preferences, clinicians were less likely to initiate contact owing to fears of intruding (CMOC5).^9,31,32,34^

### Bereaved patients' expectations

Most patients expected proactive contact from their GP following a bereavement — described as a telephone call, letter, or other acknowledgement — and viewed this as part of the clinician’s job (CMOC7).^45–48^ By contrast, some patients felt GP involvement in bereavement was not relevant,^48^ and others reported there was ‘no point’ discussing bereavement with their GP (CMOC8, 9).^49^

What shaped patient expectations was unclear: one study indicated that expectations were informed by how patients viewed the role of the GP, and their perceived relationship with their GP.^46^ Some patients described feeling unable to approach their doctor owing to feeling overwhelmed by grief,^50–52^ and for certain types of death, feelings of shame and stigma discouraged disclosure to their GP (CMOC11, 12).^48,53^ Patients expressed worry about wasting the GP’s time or acting as a ‘nuisance’.^47^ Further, patients were concerned that other health complaints might be ‘trivialised’ as part of a grief reaction,^45^ and described fears of being given medication for their bereavement when instead they sought a listening ear or information (CMOC9).^48^

If GPs were not contacted by patients, they would assume that the patient was coping (CMOC10).^28,31,40^ When patients took the initiative to contact, GPs took these requests more seriously.^31^

Initial support was offered in most cases but longer term GPs took a reactive role.^10,35–37^ GPs assumed social support networks were generally the most appropriate means of bereavement support, and a minority of patients also held this view (CMOC9, 10). Professional intervention was viewed as something that might ‘weaken’ informal support networks.^35–37,40^

### Responding to bereaved patients

Clinicians reported feeling unprepared to manage bereavement.^32,40,54–56^ Even with training, clinicians reported low confidence.^24^ Knowledge of contemporary theories of grief was low, and outdated stage models of grief were often utilised.^27,28,40,57^ GPs’ ability to identify CG risk factors was poor,^25^ but agreed that sudden death, previous mental health history, and death of a child presented high risk of complications (CMOC13).^27,40,50^

Knowledge gaps concerning local bereavement services and where to refer patients was reported, causing delayed or inappropriate referrals,^58^ and an increased likelihood of prescribing medication.^9,25,50,51^ Some clinicians were hesitant to refer if they believed that further support for bereavement was not useful (CMOC15, 16).^40^

For some, personal experiences of bereavement and/or cultural or religious beliefs were more influential than formal training (CMOC14).^33,35,40,59^ Clinicians with personal bereavement experience reported increased confidence in dealing with bereaved patients and improved empathy and understanding (CMOC19).^32,34,60^ Clinician interest in palliative care and bereavement increased with age.^32,35^ Personal loss also increased confidence in diagnosing PGD.^24^

Clinicians reported finding bereavement care emotionally challenging (CMOC17).^9,27,33,34,59,61^ Distressed patients could cause discomfort and feelings of helplessness.^9,33,34^ Perceived comfort levels with crying were associated with clinician responsiveness to the patient’s grief.^27^ Clinicians identified with the bereaved patient, for instance, in the type of death and/or family circumstances, which both increased attentiveness and provoked emotional responses (CMOC20).^9,34,35,42,59^ Clinicians also experienced grief toward deceased patients and at times guilt or regret, which fostered wariness about bereavement care involvement (CMOC21).^9,28,33,35,59^

For patients, the opportunity to speak about their grief and to be listened to was important (CMOC18, 21).^27,43,46–48,53,62^ Clinician avoidance of discussing the loss was viewed as negative and dismissive,^48^ whereas displaying emotions, being attentive to patient feelings, and, in some cases, physical touch were viewed positively.^46^ Perceived negative responses from clinicians could increase difficulties in the grieving process (CMOC18)^25,40^ .

## Discussion

### Summary

Bereavement care is considered an important part of primary care; however, provision remains inconsistent and clinicians experience ambiguities concerning the appropriate extent of their involvement with bereaved patients. Practitioners encounter practical challenges in terms of not being aware about deaths, but bereavement can also be confronting as practitioners, at times, struggle to support distressed grieving patients and manage their own emotions.

The clear education and training needs notwithstanding, clinicians described feeling unprepared, and at times unsupported to manage bereaved patients. Positive attributes of general practice, particularly the trust and established relationships that come with having a continuity of care and/or having an understanding of patient preferences and needs were described as beneficial in knowing how and when to approach patients following bereavement.

In terms of primary care management of CG or PGD, a lack of literature was found; further research and improved training and education to guide primary care practice are needed in this area.^50,63^

### Comparison with other literature

Issues facing primary care raised by this review are not new, and debates over the appropriate role of the GP in bereavement care appear little changed from over 20 years ago.^8,64,65^ A previous review of bereavement care in primary care^10^ found that, although clinicians see bereavement care as an important part of their work, they receive little training and variation exists in practice. Only 15 articles published since 2011 were identified as relevant, indicating that evidence in this area remains limited. A US review reported similar limitations.^63^

Review findings have unearthed the importance of attitudes, assumptions, and expectations of bereavement care held by both clinicians and patients, suggesting an attitudinal component that needs addressing in interventions for both general bereavement and for CG or PGD.^66^ While mental health care is well integrated into general practice, clinician attitudes towards bereavement care appear hesitant. Clinical intervention into bereavement remains controversial across a number of specialties, including general practice.^66–69^ PGD has only recently been recognised as a clinical diagnosis; with increased training and awareness among primary care clinicians, attitudes and understanding of CG and PGD may improve. Nonetheless, bereavement is an intensely painful experience with no rapid or easy solution; addressing the support needs of bereaved people might be perceived as a time-consuming prospect. Underlying this is a concern of medicalising a social experience, creating a need for health services to respond.^35^ However, maintaining that bereavement is solely the responsibility of social networks and community is not consistent with the holistic and compassionate care that characterises general practice.

Relying on patients to seek care, encouraged in recent UK policy changes,^70^ may be inappropriate for bereavement. Presently, bereaved patients and clinicians appear to have conflicting views on whose responsibility it is to initiate contact; in assuming that patients are coping, clinicians may be unaware that many of their patients want and expect some form of support.^71^

Grief is in many ways an ‘invisible condition’^72^ and discussing bereavement experiences can be challenging for patients.^73^ Public awareness of complicated and prolonged grief is low, and susceptible to negative perceptions.^74^ Clinicians also struggle to know how to communicate with bereaved patients^75^ and how to address their needs.^76^ Education needs are apparent and while personal experiences could compensate for a lack of formal training,^77^ such experiences should not be used as a substitute for an understanding of the research evidence.

### Strengths and limitations

The realist approach has drawn out potential mechanisms that deepen understanding of CG and bereavement in primary care. A further strength is the inclusion of a broad range of literature.

The evolving definitions and terminology of CG and PGD has consequences when evaluating the practical application of the evidence. With the use of different terms, it cannot be assumed the same condition is referred to across the literature.

The screening and selection of articles was carried out by one researcher. Decisions were regularly shared with the team to address potential biases; however, it is acknowledged that relevant articles or data may have been missed.

There have been changes in policy and practice since publication of the earlier studies included in the review, and the applicability of these findings needs to be considered within the current context.

### Implications for research and practice

The authors recommend bereavement care is recognised as a legitimate part of primary care and understood as a community response (see Box 2). Response to bereavement should take a tiered, targeted approach.^78^

Box 2 **Implications for improving bereavement care in primary care practice**

**Table:** 

Consider developing and implementing local strategies for death notification, which might include a death register (CMOCs 1, 2, 3) Seek an understanding of individual patient preferences: what type of bereavement support does a patient want? (CMOCs 5, 6) Initiate contact when patient bereavement is known; for example, by a phone call or condolence letter (CMOCs 7, 8, 9, 10, 11, 12) When contacting patients include details about available local support services and invite them to make an appointment if they are experiencing problems (CMOCs 8, 9, 12) Where appropriate, utilise link workers to help connect patients with local support services or community groups (CMOCs 8, 9, 12, 16) When consulting with bereaved patients utilise active listening skills: feeling listened to may be of the most value to patients (CMOCs 18, 21) Revising medical education and training to include up-to-date grief theories. This could include, for example, bereaved people speaking about their experiences (CMOCs 13, 14, 17, 19, 20, 21) Further training such as a short course to help clinicians who may need support with their own experiences of bereavement when managing a patient (CMOCs 13, 17, 19, 20, 21)

A community response to bereavement focuses on developing the existing ‘assets’ of community resources and creating ‘compassionate communities’, which includes both professionals and local informal caring networks.^79,80^ Social prescribing may be an appropriate means to offer bereavement support in UK primary care.^81^ Link worker roles could be utilised to connect patients with local services and community groups. This may allow GPs to focus attention to those with prolonged and complicated grief who may need medical attention. Primary care networks might usefully consider developing specialist bereavement services across their larger numbers of patients.

Significant knowledge gaps remain that have implications for practice and warrant further research. Targeted interventions for those experiencing CG is most effective,^82,83^ yet at present evidence on what, how, and for whom such treatments work is limited.^15,82^ Interventions such as condolence letters and death registers have been subject to little empirical evaluation.^84^ The efficacy of education recommendations also remains largely unknown.^25^

Primary care services are well-placed to provide bereavement support, but clinicians may be reluctant to take a proactive role. Bereavement can bring challenging emotions and feelings for both bereaved patients and for the clinicians managing their care. This hesitancy to be involved in bereavement is particularly concerning as primary care faces the ongoing impact of the COVID-19 pandemic, and a likely increase of bereaved patients in need of support. A community level response to bereavement that tailors support according to risk is needed to ensure that neither clinicians nor patients are left to manage the burden of bereavement, and to ensure that bereavement care becomes a standard part of practice.
